# hUMSCs regulate the differentiation of ovarian stromal cells via TGF-β_1_/Smad3 signaling pathway to inhibit ovarian fibrosis to repair ovarian function in POI rats

**DOI:** 10.1186/s13287-020-01904-3

**Published:** 2020-09-07

**Authors:** Linlu Cui, Hongchu Bao, Zhongfeng Liu, Xuejing Man, Hongyuan Liu, Yun Hou, Qianqian Luo, Siyuan Wang, Qiang Fu, Hongqin Zhang

**Affiliations:** 1 College of Basic Medicine & Institute of Reproductive Diseases, Binzhou Medical University, Yantai, 264003 Shandong China; 2 College of Basic Medicine, Binzhou Medical University, Yantai, 264003 Shandong China; 3 Department of Clinical Medicine, Yantai Yuhuangding Hospital, Yantai, 264000 Shandong China; 4 Yantai Affiliated Hospital of Binzhou Medical University, Yantai, 264100 Shandong China; 5 Clinical Medical School, Binzhou Medical University, Yantai, Shandong China; 6 School of pharmacy, Binzhou Medical University, Yantai, Shandong China

**Keywords:** Primary ovarian insufficiency, Human umbilical cord-derived mesenchymal stem cells, Ovarian stromal cells, TGF-β_1_ signaling pathway, Fibrosis

## Abstract

**Objective:**

The basic pathological changes of primary ovarian insufficiency (POI) include ovarian tissue fibrosis and follicular development disorders. The human umbilical cord mesenchymal stem cell (hUMSC) transplantation has been shown an effective method to improve the ovarian function in POI rat model; however, the exact mechanisms are still unclear. The purpose of this study is to investigate whether the recovery of ovarian function in POI rats is related to the inhibition of tissue fibrosis following hUMSC transplantation. Furthermore, the transforming growth factor-β_1_ (TGF-β_1_) signaling pathway is explored to determine the mechanisms of ovarian function recovery through its inhibition of tissue fibrosis.

**Methods:**

The primary ovarian insufficiency (POI) rat model was established by intraperitoneal injection of chemotherapy drug cisplatin (CDDP) for 7 days. The levels of serum sex hormones were measured using enzyme-linked immunosorbent assay (ELISA). The tissue fibrosis in the ovary was examined using Masson staining and Sirius red staining. The collagen fibers in the ovarian tissues were detected by Western blot analysis. To investigate the mechanisms of ovarian function recovery following hUMSC transplantation, ovarian stromal cells were isolated from the ovarian cortex of immature rats. The expression of Cytochrome P450 17A1 (Cyp17a1) and fibrosis marker of alpha smooth muscle actin (α-SMA) in ovarian stromal cells was examined using immunofluorescence analysis. Also, the protein levels of Cyp17a1 and α-SMA in ovarian stromal cells were examined by Western blot analysis. The expression of TGF-β_1_ and Smad3 signals was measured by Western blot and quantitative reverse-transcription polymerase chain reaction (qRT-PCR) analysis.

**Results:**

The results show that the function of the ovary in POI rats was significantly improved after hUMSC transplantation. The expression of fibrosis markers (α-SMA) and production of Collagen Type I (Collagen I) and Collagen Type III (Collagen III) in POI rats were significantly inhibited in POI rats following hUMSC transplantation. In the cultured ovarian stromal cells, the decrease of TGF-β_1_ and p-Smad3 protein expression was observed in hUMSC-treated POI rats. The treatment with TGF-β_1_ inhibitor of SB431542 further confirmed this signal pathway was involved in the process.

**Conclusion:**

Our study demonstrated that the TGF-β_1_/Smad3 signaling pathway was involved in the inhibition of ovarian tissue fibrosis, which contributed to the restoration of ovarian function in POI rats following hUMSC transplantation.

## Background

Primary ovarian insufficiency (POI) is defined by the loss of ovarian function before the age of 40 in women, which causes infertility and premenopausal syndrome [1, 2]. The clinical manifestations are secondary or primary amenorrhea for at least 4 months and characterized by a decrease in serum estradiol (E_2_) levels and an increase in gonadotropin levels [3, 4]. The basic pathological changes include ovarian tissue fibrosis and follicular development disorders, but the etiology and pathogenesis of POI are still unknown [5, 6]. There are many reports that chemotherapy as a commonly used treatment for female tumor patients may lead to ovarian failure, including follicular loss, vascular damage, and tissue fibrosis, especially in young female patients [7–10]. Therefore, it is urgent to find an effective treatment.

For the patients with primary ovarian insufficiency, the hormone replacement therapy (HRT) is the most commonly used treatment in clinical practice. Even though this treatment may alleviate the symptoms of patients with POI, it cannot fundamentally improve ovarian function. Moreover, the patients need to take medicine for a long time which may increase the risks of breast cancer and endometrial cancer [11]. Recently, the studies have shown that transplantation of mesenchymal stem cells (MSCs) could restore ovarian structure and function in POI animal models [12, 13]. The lower immunogenicity, higher proliferation capacity and less ethically controversial extraction methods to isolate the hUMSCs might serve as a promising approach for the stem cell transplantation therapy [14–16]. However, the exact mechanisms to protect ovarian function following the hUMSC treatment have not been fully investigated. During the process of follicular development, the follicular stromal cells play various necessary roles such as multiplying and differentiating into inner theca cells (TCs) to maintain the integrity of the follicle structure and outer myofibroblast (MFB) to secrete extra-cellular matrix (ECM) including collagen fibers of type I and type III, promoting the formation of follicular capillaries, which provide nutrients for follicular growth and development and thereby affecting the development and atresia of follicles [17–19]. However, the role of stromal cells in the primary ovarian dysfunction is still unclear. The current studies on ovarian stromal cells have mainly focused on its role in polycystic ovary syndrome (PCOS). Since the ovarian stromal cells can differentiate into TCs or MFB which are closely related to fibrosis of ovarian tissues, our study has focused on the effect of hUMSC transplantation on the proliferation and differentiation of ovarian stromal cells in POI rats.

TGF-β_1_ is a member of TGF-β superfamily, which includes several groups of highly conserved multifunctional cell-cell signaling proteins of key importance in the control of cell growth, differentiation, embryogenesis, and immune suppression and repair after injury [20]. Some studies showed that the TGF-β signaling pathway mediated mainly by Smad proteins and TGF-β_1_/Smad3 signaling pathway has been involved in the pathogenesis of renal fibrosis [21, 22]. During the renal fibrosis, the mesenchymal cells, intrinsic cells, and infiltrating inflammatory cells in the renal all produced TGF-β_1_, which leads to the transformation of fibroblasts in the stroma into MFB and the formation and deposition of ECM [23]. Similarly, the basic pathological changes of POI are tissue fibrosis. And in ovarian tissue, stromal cells can proliferate and differentiate into membrane cells or MFB. MFB can synthesize and secrete extracellular matrix, which may lead to organ fibrosis when there is too much extracellular matrix. Therefore, the goal of the current study is to investigate whether the hUMSC transplantation could inhibit ovarian fibrosis development in POI rats and further explore its mechanisms to see if TGF-β_1_/Smad3 signaling pathway is involved in this process.

## Materials and methods

### Animals

The female Wistar rats (*n* = 120) at the age of 7 weeks were purchased from the Jinan Pengyue Experimental Animal Breeding, Co., Ltd. (Shandong, China) for in vivo experiments. Female Wistar rats aged 3–4 weeks (*n* = 40) were purchased from the Jinan Pengyue Experimental Animal Breeding, Co., Ltd. (Shandong, China) for in vitro experiments. The rats were housed in a cage under reference atmospheres and free access to food and water. All the experimental procedures have been approved by the Institutional Animal Care and Use Committee at Binzhou Medical University. The study was conducted in accordance with the National Research Council Guidance for Care and Use of Laboratory Animals.

### Chemicals

Cisplatin (CDDP) (Meilunbio, China) was dissolved in warmed distilled water (DW) at a stock concentration of 3.33 mM and added to stromal cell cultures at a final concentration of 0–60 μM. The TGF-β_1_ inhibitor, SB431542 (Selleck, USA), was dissolved in DMSO at a stock concentration of 10 mM and added to stromal cell cultures at a final concentration of 10 μM.

### Group and treatment

According to reports in the literature, CDDP as a common anticancer drug can lead to POI [12, 24]. The POI model was generated by daily intraperitoneal injections of CDDP (2 mg/kg, dissolved in saline) for 7 days [25, 26]. The rats were randomly assigned to four groups: control, POI + hUMSCs, POI, and POI + PBS group. In the control group, normal rats were injected intraperitoneally with physiological saline for 7 days; in the POI + hUMSC group, 7 days after the injection of CDDP, 200 μl phosphate-buffered saline (PBS) containing 2 × 10^6^ hUMSCs were injected into the tail vein of POI rats; in the POI group, the rats were injected intraperitoneally with CDDP for 7 days; in the POI + PBS group, the POI rats were injected with 200 μl PBS via the tail vein. After 1-week treatment, 14 rats were randomly selected from each group for fertility testing. The remaining rats were necropsied for the study.

### Isolation and culture of hUCMSCs

The umbilical cords were collected from healthy donors who received cesarean section and signed a written informed consent. The collected umbilical cord was washed twice with PBS, mechanically minced, and then cultured with low-glucose Dulbecco’ s modified Eagle’ s medium containing (Gibco) supplemented with 10% fetal bovine serum (FBS, Gibco, South America), 1% 100 U/mL streptomycin sulfate, and 100 U/mL penicillin G in a humidified atmosphere with 5% CO_2_ at 37 °C. To confirm the phenotype of hUMSCs, cell morphology was observed under a light microscope. The differentiation ability to adipocytes and osteoblast was examined by alizarin red staining and oil red O staining. The cell surface and intracellular markers such as CD44, CD73, CD90, CD34, HLA-DR, CD45, and CD105 were examined using flow cytometry (FCM). The cells at the third to fifth generation were selected for the experiments; the culture supernatant of hUMSCs was also collected. The supernatant collected from different batches were mixed evenly and stored separately for subsequent experiments.

### Isolation, culture, and identification of ovarian stromal cells

Ovarian stromal cells were isolated from the ovarian cortex of normal immature 3- to 4-week-old rats. Briefly, the surface epithelium and medulla of the ovary were removed. The GCs were isolated by puncture of follicles with sterile syringes under a stereoscope (Olympus, Japan). The remaining ovary tissues were cut into 1 mm^3^ fragments using scissors and washed with PBS for three times. The tissues were digested with type II collagenase that was dissolved in McCoy’ s 5A (Modified) Medium at 37 °C for 60 min. After digestion, the dispersed cells were washed with McCoy’ s 5A medium and centrifuged at 1000 rpm at 37 °C for 5 min. The pellet was suspended in McCoy’ s 5A medium containing 10% FBS (AusGeneX, Australia), 1% 100 U/mL streptomycin sulfate, and 100 U/mL penicillin G and cultured in a 37 °C humidified incubator with 5% CO^2^.

### Fertility examination

Fourteen rats were randomly selected from each group including control, POI + hUMSCs, POI, and POI + PBS group. The female rats were mated at 2:1 ratio with sexually mature male rats. The vaginal plug was observed at 08:00 every morning to determine the mating is successful or not. After vaginal plugs appeared, they were euthanized according to experimental needs to determine pregnancy status and observe the pregnancy rate. And the uterus was collected and examined for the number of developed fetuses.

### Enzyme-linked immunosorbent assay (ELISA)

The serum of each rat was collected and stored at − 80 °C after centrifugation for analysis. The serum levels of estradiol (E_2_), follicle-stimulating hormone (FSH), and luteinizing hormone (LH) were measured using ELISA kit following the manufacturer’s instructions (Mlbio, China).

### Hematoxylin and eosin (H&E) staining

The ovaries were collected from the rats of each group and fixed in 4% paraformaldehyde for 24 h. Then, the tissues were processed by paraffin embedding, sectioning (5 μm), and hematoxylin and eosin (H&E) staining. To analyze the ovarian morphology and count the numbers of ovarian follicles, the slides were examined under a light microscope. The follicles were categorized as primordial follicles, primary follicles, secondary follicles, and atresia follicles according to the previous report [27].

### Masson trichrome staining and Sirius red staining

The ovaries were collected from the rats of each group and fixed in 4% paraformaldehyde for 24 h. Following the paraffin embedding and sectioning (5 μm) process, the tissues were stained with Masson trichrome and Sirius red. To evaluate the fibrosis of the ovary tissues in each group, the slides were analyzed and photographed under a light microscope. Five fields in each staining image were randomly selected for examination. ImageJ software was used to quantitate the degree of interstitial fibrosis in the ovary tissue.

### CCK-8 cell viability assay

The effect of CDDP and TGF-β_1_ inhibitor of SB431542 at different concentrations on the viability of stromal cells was measured using CCK-8 kit (Meilong Bio, China). The cells (5000 stromal cells/well) were seeded in 96-well plates and incubated overnight. After cell adhesion, the medium is replaced with media containing CDDP (0–60 μm) or SB431542 (0–40 μm). CCK-8 cells (10 μl) were added to the cell medium for 1 h. The absorbance was measured at 450 nm using an ELISA assay kit according to the manufacturer’s instructions.

### Inhibitor experiment

After achieving 80% confluence, stromal cells were seeded in 6-well plates (1 × 10^5^ cells/well). Cells were divided into four groups according the different treatments for 20 h: (1) control group-untreated medium, (2) CDDP group − CDDP (20 μM) alone, (3) CDDP + hUMSC group − CDDP + hUMSC supernatants, and (4) CDDP + SB431542 group − CDDP + SB431542 (10 μM).

### Immunofluorescence staining

The expression of vimentin, Factor VII, Cytokeratin, Cyp17a1, and α-SMA proteins was detected by immunofluorescence staining. Ovarian stromal cells were washed three times with PBS and fixed in 4% paraformaldehyde for 20 min. After washing, the cells were blocked for 30 min in PBS containing 5% donkey serum (Santa Cruz Biotechnology). To identify the mesenchymal, epithelial, endothelial cells, TCs, and MFB, the cells were incubated with the primary antibody of anti-vimentin (1:100, Proteintech, China), anti-cytokeratin (1:100, Proteintech, China), anti-Factor VIII (1:100, Proteintech, China), and the mixture of anti-Cyp17a1 (1:100, Abcam, UK) and anti-α-SMA (1:100, Abcam, UK) at 4 °C overnight, respectively. After rinsing, the stromal cells were incubated with the second antibody of goat anti-rabbit IgG, Alexa Fluor 488 (Invitrogen, USA), goat anti-rabbit IgG, goat anti-rabbit IgG, Alexa Fluor 596 (Invitrogen, USA), and the mixture of goat anti-rabbit IgG, Alexa Fluor 488, and goat anti-mouse IgG, Alexa Fluor 596 (Invitrogen, USA), respectively at 37 °C for 1 h with DAPI (Solarbio, China) staining solution. The staining of the cells was visualized using a fluorescent microscope (Leica, Germany).

### Quantitative reverse-transcription polymerase chain reaction (qRT-PCR)

Total RNA was isolated from ovarian stromal cells using Trizol reagent (Ambion, USA) and reversed transcribed into cDNA using Transcriptor HiFi cDNA Synth (Roche, Germany). The primers for quantitative real-time polymerase chain reaction were listed as follows: transforming growth factor-β_1_ (TGF-β_1_) forward primer: CATTGCTGTCCCGTCAGA and reverse primer: AGGTAACGCCAGGAATTGTTGCTA; Smad3 forward primer: GCACAGCAAGTTCCCAGTGTGTA and reverse primer: GCCATGCATCCACTGTTCC; Cytochrome P450 17A1 (Cyp17a1) forward primer: GGCATCTCAAGCAAACACCAT and reverse primer: GCTGTGCGGATATTCAAGGAT; alpha smooth muscle Actin (α-SMA) forward primer: GGCCGAGATCTCACTGACTAC and reverse primer: TTCATGGATGCCAGCAGA; Glyceraldehyde-3-phosphate dehydrogenase (GAPDH) forward primer: GGCACAGTCAAGGCTGAGAATG and reverse primer: ATGGTGGTGAAGACGCCAGTA. The housekeeping gene GAPDH was used to normalize the gene expression. The testing in each group was repeated in triplicate.

### Western blot

For Western blotting analysis, ovary tissues and cultured stromal cells were lysed using radioimmunoprecipitation assay (RIPA) buffer and the protein concentration was measured by bicinchoninic acid assay. The samples were electrophoresed on sodium dodecyl sulfate polyacrylamide gels and transferred to a PVDF membrane. After blocking with 5% ~ 7% skim milk, the membranes were incubated with anti-TGF-β_1_ (1:1000, Abcam, UK), anti-Smad3 (1:1000, Abcam, UK), anti-p-Smad3 (1:1000, Proteintech, China), anti-Cyp17a1 (1:4000, Abcam, UK), anti-α-SMA (1:1000, Abcam, UK), anti-Collagen I (1:400, Proteintech, China), anti-Collagen III (1:400, Proteintech, China), and anti-GAPDH (1:20000, Proteintech, China) polyclonal antibodies for overnight. After incubation, the membranes were washed with TBS and Tween 20 (TBST) three times and then immunoblotted with HRP-conjugated secondary antibodies (Proteintech) for 1 h at room temperature. The expression of each protein was measured by an enhanced chemiluminescence reagent (ECL) kit (Sparkjade Science Co., Ltd., China). The density of protein expression bands was measured using ImageJ software.

### Data analysis

Measurement data were expressed as mean ± SD and analyzed by SPSS 22.0 software. Differences between two groups were determined using one-way analysis of variance (ANOVA) with post hoc Bonferroni test. And enumeration data was expressed by rate, and chi-square test was used for comparison between groups. A value of *P* < 0.05 is considered a significant difference.

## Results

### Characterization of hUMSCs

In order to confirm whether the isolated cells were hUMSCs, the examination with light microscopy, flow cytometry, osteogenesis induction, and adipocyte induction was performed. As shown in Supplementary Figure 1 j, the cells isolated from the human placenta started to form a cluster of clones after 7 to 10 days of incubation. The morphology of cells appears similar to fibroblasts. Following staining, the cellular expression of CD44 was 99.7%; in the meantime, the expression of CD90, CD73, and CD105 were 99.9%, 97.1%, and 99.7%, respectively (Supplementary Figure 1 a-c, g). In comparison, the expression of CD45, CD34, and HLA-DR was all below lower than 5% as illustrated in Supplementary Figure 1 d-f. After incubation for 28 days and 14 days, respectively, the cells showed the ability to differentiate into osteoblasts and adipocytes, which were confirmed by positive von Kossa staining and Oil Red O staining Supplementary Figure 1 h-i. These results are consistent with the previous literature reports on the phenotypic characteristics of hUMSCs [24, 28, 29].

### Effects of hUMSC transplantation on ovarian function in POI rats

**Fig. 1:**
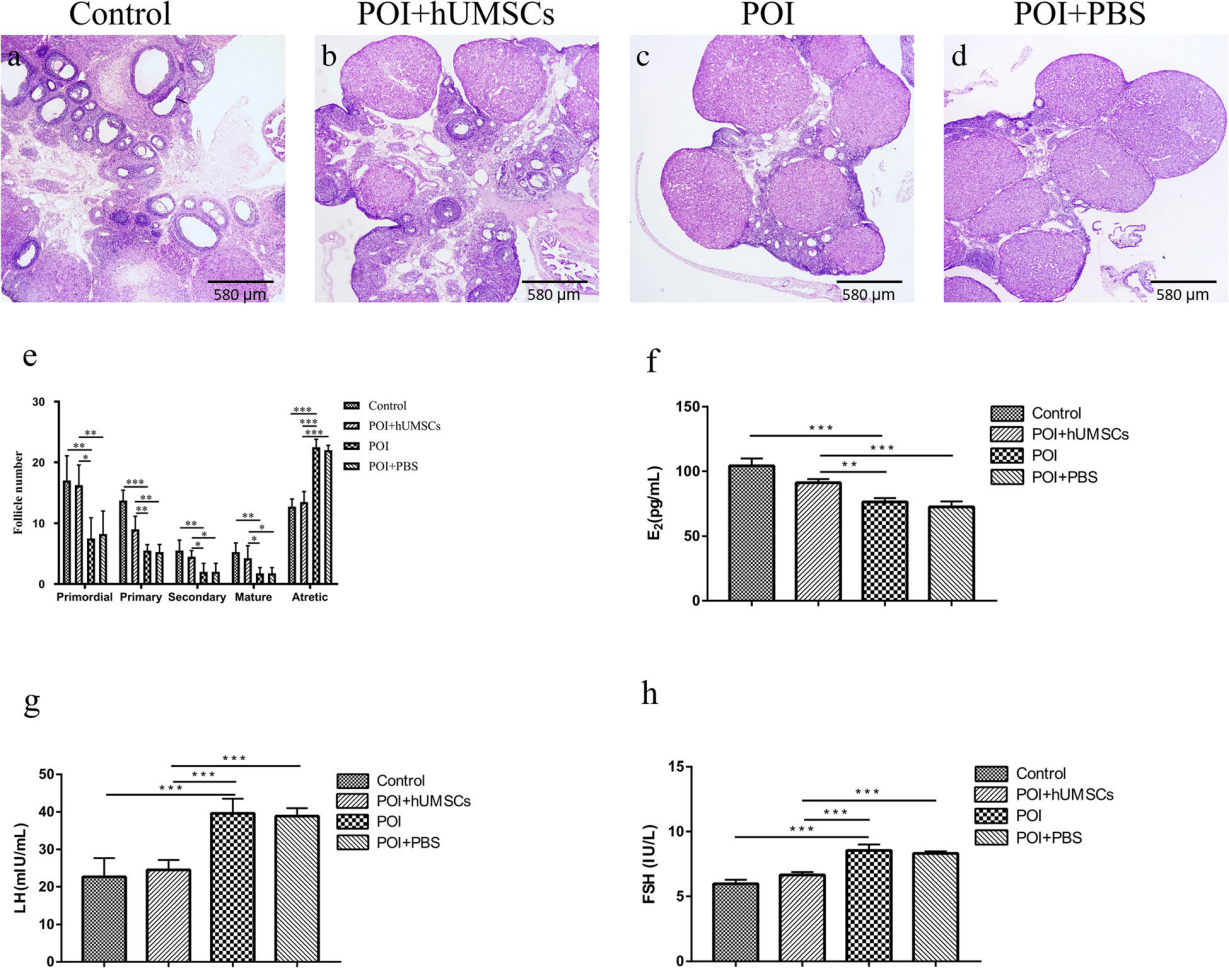
Morphological features and functional changes of the ovary. **a**–**d**, ×40: The ovarian tissue was examined by H&E staining in each group. **e** Summary of follicle number changes in the ovary of each group. **f**–**h** Serum levels of E_2_, LH, and FSH hormones in each group. Data are expressed as the means ± SD. *n* = 40, *P < 0.05, **P < 0.01, and ***P < 0.001. HE hematoxylin and eosin, E_2_ estradiol, LH luteinizing hormone, FSH follicle-stimulating hormone, and SD standard deviation

To explore the effects of hUMSC transplantation on ovarian function in POI rats, the histological pathology of ovarian tissues, the number of follicles at each developmental stage, and the serum levels of sex hormones in the rats were examined. As shown in Fig. 1a–d, the control group showed a large number of healthy follicles at each stage including primordial follicles, primary follicles, secondary follicles, and antral follicles. In contrast, the ovarian tissue atrophy and the decrease of follicles number at each developmental stage were observed in POI rats compared to the control group (Fig. 1e). Following hUMSC transplantation, the number of developing follicles including primordial follicles, primary follicles, secondary follicles, and antral follicles in the POI + hUMSC group was significantly higher than POI + PBS group (Fig. 1e). For the serum hormone measurement, it is noted that the lower level of E_2_ and higher levels of FSH and LH in POI rats compared to the control group (Fig. 1f–h). However, 1 week after the transplantation of hUMSCs, the serum E_2_ levels were significantly increased and the FSH and LH secretion were decreased in POI rats as summarized in Fig. 1f–h. Overall, the data showed that the hUMSC transplantation helps restore the ovarian function which was damaged by CDDP in POI rats.

### Effects of hUMSC transplantation on ovarian fibrosis in POI rats

**Fig. 2:**
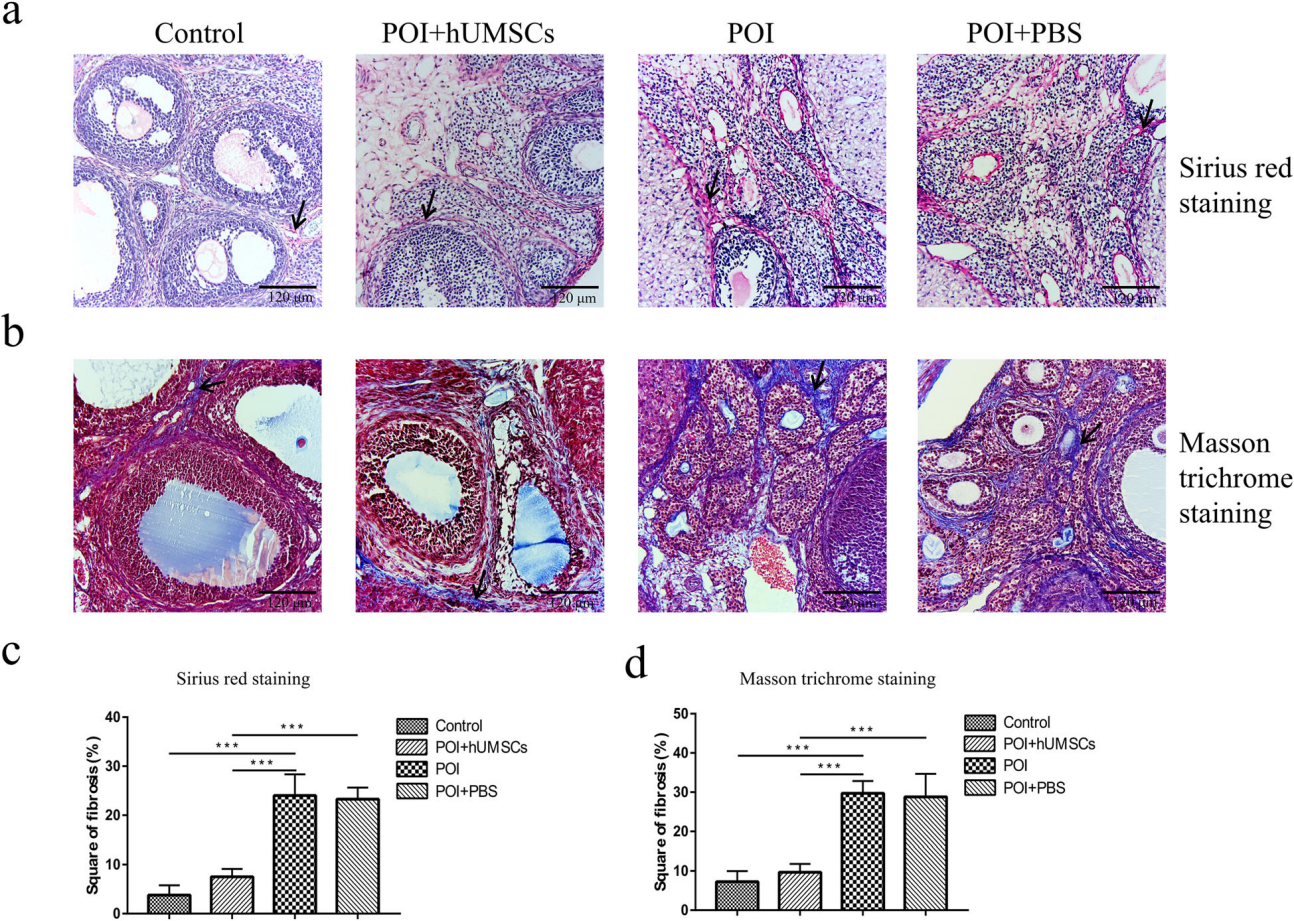
Ovarian fibrosis examination by histopathological analysis. **a**, **b**, ×200]: Sirius red staining and Masson trichrome staining of the ovarian tissue in each group observed under a microscope. Arrows indicate staining of type I and type III collagen fibers. **c**, **d** The score of stained Sirius red and Masson trichrome Staining was quantitated using ImageJ software. The value is expressed as the mean ± SD. *n* = 40, *P < 0.05, **P < 0.01, and ***P < 0.001. SD standard deviation

**Fig. 3:**
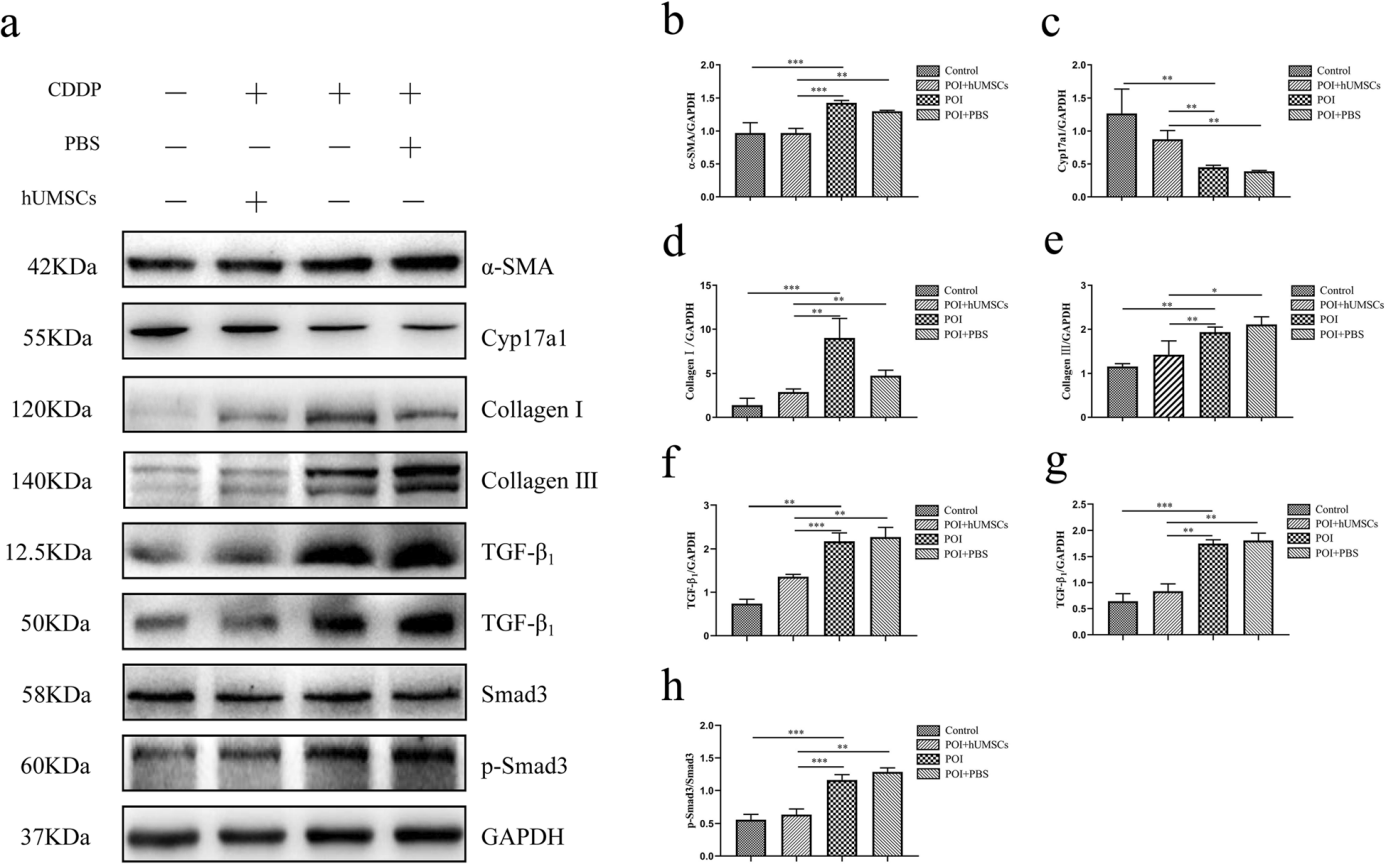
Analysis of ovarian tissue fibrosis-related protein by Western blot. **a** The expression of α-SMA, Cyp17a1, Collagen I, Collagen III, TGF-β_1_, Smad3, and p-smad3 in the ovarian tissues of each group. A representative blot from one of three independent experiments is shown. GAPDH was used as the loading control. **b**–**h** The expression of α-SMA, Cyp17a1, Collagen I, Collagen III, TGF-β_1_, Smad3, and p-smad3 protein was quantitated using Image J software. Data are expressed as the means ± SD. *n* = 20, *P < 0.05, **P < 0.01, and ***P < 0.001. α-SMA alpha smooth muscle actin, Cyp17a1 Cytochrome P450 17A1, Collagen I Collagen Type I, Collagen III Collagen Type III, TGF-β_1_ transforming growth factor-β_1,_ GAPDH Glyceraldehyde-3-phosphate dehydrogenase, and SD standard deviation

In order to investigate if hUMSC transplantation can reduce the ovarian tissue fibrosis in POI rats, the tissues were stained with Sirius red and Masson trichrome. Also, the Western blot analysis was performed on the collected ovarian tissues. As shown in Fig. 2a, b, the staining results showed the increase of fibrotic tissues in the ovary of POI rats when compared to the control group. After the hUMSC transplantation, the fibrosis area in the ovarian tissues of POI rats was significantly reduced. In addition, the results from the Western blot analysis showed that the expression of TC marker of Cyp17a1 [30, 31] was significantly decreased in POI rats as shown in Fig. 3c. However, the expression of MFB marker α-SMA [32] was increased in POI rats. The amounts of Collagen I and Collagen III were significantly decreased in POI rats following hUMSC transplant (Fig. 3d–e). In addition, the results showed that POI + hUMSC group compared with POI + PBS group, the expression of TGF-β_1_ and p-smad protein decreased. Collectively, these data indicated that the hUMSC transplantation effectively inhibited the fibrogenesis of ovarian tissues in POI rats. And this effect is probably related to the TGF-β_1_ signaling pathway (Fig. 3f–h). These pathology changes contribute to the restoration of the ovarian function and the development of ovarian stromal cells into membrane cells in CDDP-induced POI rats.

### Effects of hUMSC transplantation on the fertility of POI rats

**Fig. 4:**
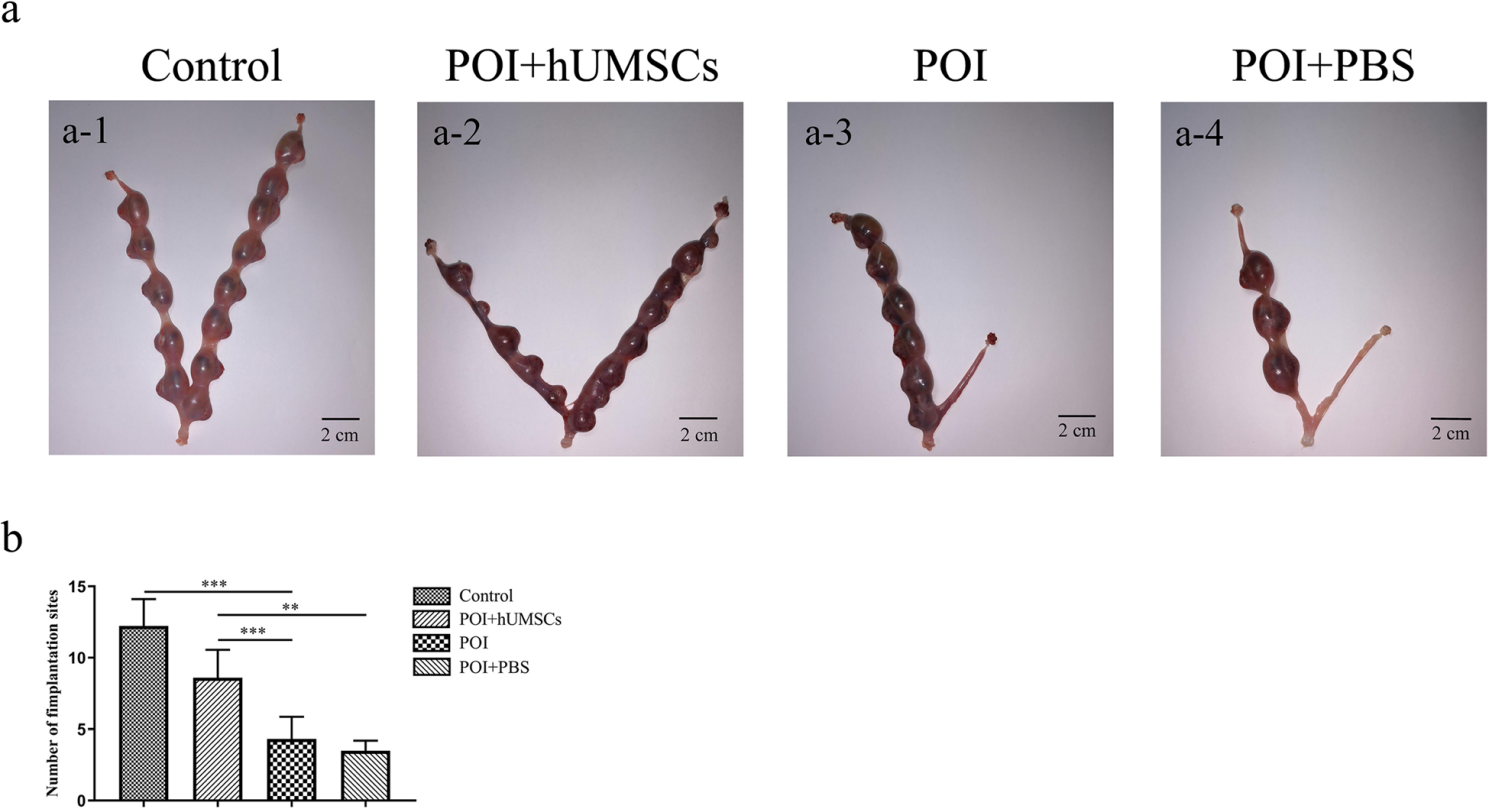
Effects of hUMSC transplantation on the fertility of POI rats. **a** Gross observation of ovarian specimen in each group. **b** Summary of embryo numbers at implantation sites of each group. Data are expressed as the means ± SD. *n* = 56, *P < 0.05, **P < 0.01, and ***P < 0.001. hUMSCs human umbilical cord-derived mesenchymal stem cells, POI primary ovarian insufficiency, and SD standard deviation

To explore the effect of hUMSC transplantation on the fertility of POI rats, the embryo-implantation efficiency and pregnancy rate were examined in the rats of each group. As shown in Fig. 4, the efficiency of embryo implantation in POI group was significantly lower than the control group. However, the hUMSC implantation significantly increased the efficiency of embryo implantation in POI rats. In addition, the results from Table 1 showed that the pregnancy rate of POI rats was significantly increased after hUMSC transplantation. In summary, the above data suggested that hUMSC transplantation can help restore the fertility of POI rats.

**Table 1:** Effect of hUMSC transplantation on pregnancy rate of POI rats. hUMSC human umbilical cord-derived mesenchymal stem cells

Group	Pregnancy	Not pregnancy	Pregnancy, rate %(*n*/*n*)	*λ*^2^ value	*P* value
Control	13	1	92.9(13/14)	14.583^a^	0.000^a^
hUMSCs + POI	9	5	64.3(9/14)	5.25^b^	0.022^b^
POI	3	11	21.4(3/14)		
POI + PBS	2	12	14.3(2/14)		

Notes: Pregnancy rate = total number of pregnancy/total number of matings

^a^Control group versus POI group

^b^hUMSCs + POI group versus POI group

### Effects of hUMSC medium on stromal cell differentiation in cultured cells

**Fig. 5:**
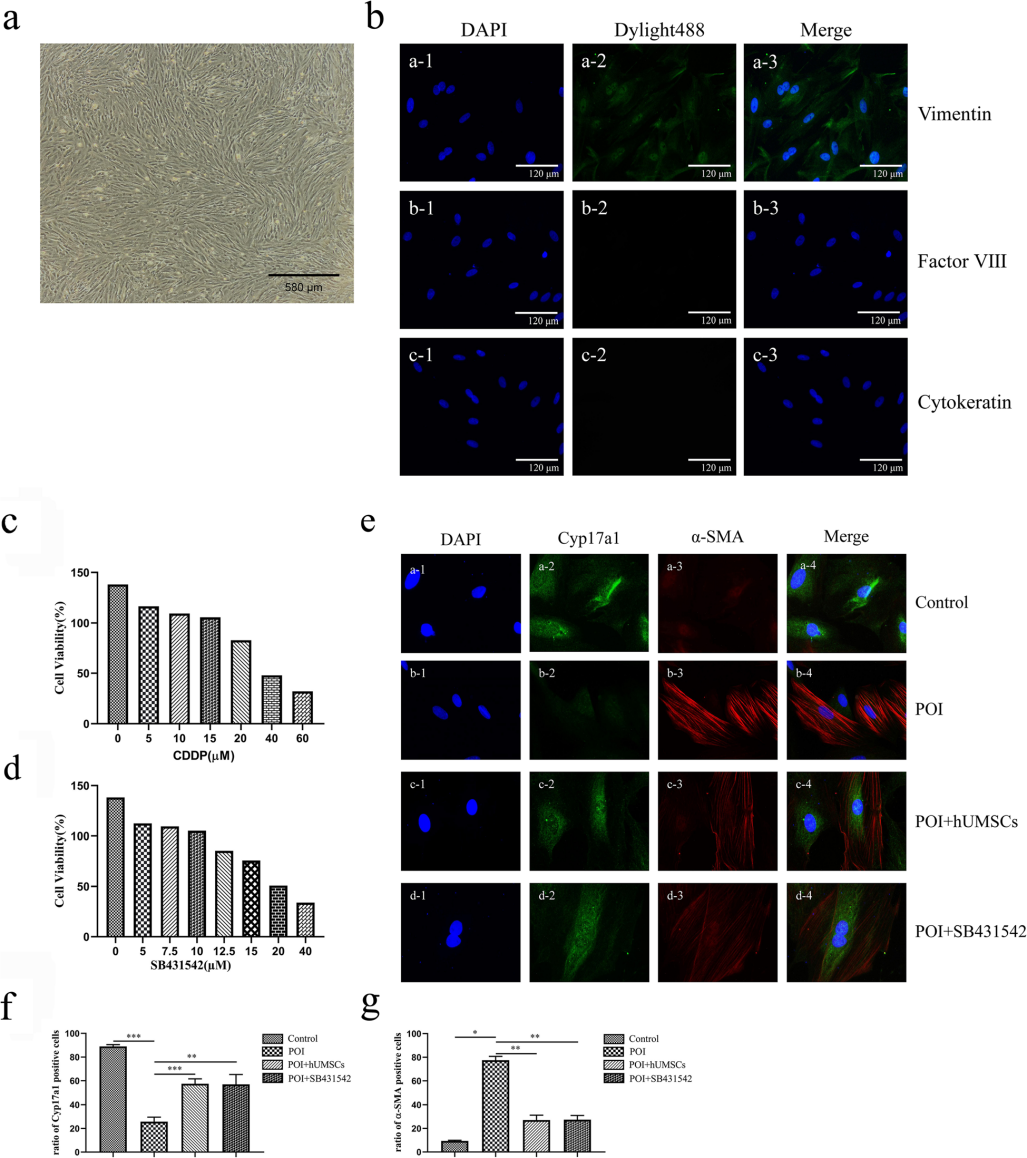
Theca cell differentiation and fibrosis expression in cultured ovarian stromal cells. **a**, ×40: The morphology of stromal cells under a microscope. **b**, ×200: The isolated cells were characterized with the immunofluorescence. **b** a1–a3: The mesenchymal cells were stained by Vimentin as green. **b**, b1–c3: The endothelial cells were stained with Factor VIII as red, and the epithelial cells were stained with cytokeratin as red. The cell nucleus was stained by DAPI as blue. **c**, **d** Cell viability of stromal cells was measured after different concentrations of CDDP or TGF-β_1_ inhibitor SB431542 treatment. **e** The expression of Cyp17a1 and α-SMA in stromal cells by immunofluorescence staining. The nucleus was stained by DAPI as blue. The theca cells were stained with Cyp17a1 as green. The MFB were stained with α-SMA as red. **f**–**g** Quantitative intensity of Cyp7a1 and α-SMA staining in each group. Data are expressed as the means ± SD. *P < 0.05, **P < 0.01, and ***P < 0.001. CDDP cisplatin, Cyp17a1 Cytochrome P450 17A1, α-SMA alpha smooth muscle actin, DAPI 4,6-diamino-2-phenyl indole, and MFB myofibroblast

**Fig. 6:**
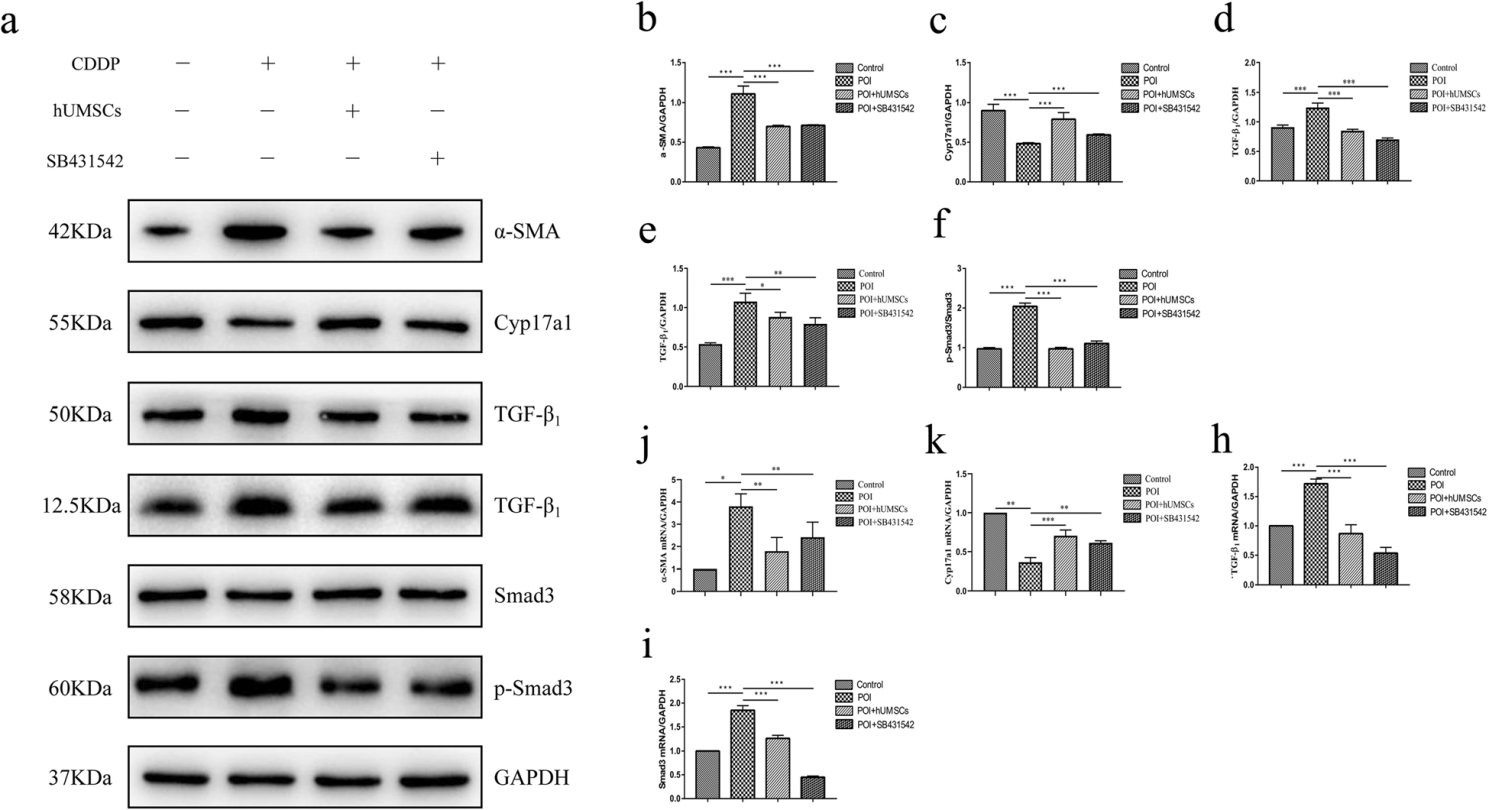
Analysis of protein and RNA expression of cell differentiation following the treatment of TGF-β_1_ inhibitor in cultured stromal cells. **a** Representative blot of Western blot analysis on each cell differentiation and fibrosis marker in hUMSC medium-cultured stromal cells with and without SB431542 treatment. **b**–**f** Quantitation on protein expression of cell differentiation marker α-SMA, Cyp17a1, and TGF-β_1_/Smad3 signaling pathway in each group. **g**–**i** The RNA expression of α-SMA, Cyp17a1, TGF-β1, and Smad3 by qRT-PCR analysis in each group. GAPDH was used as an internal control. Data are expressed as the means ± SD. *P < 0.05, **P < 0.01, and ***P < 0.001. TGF-β_1_ transforming growth factor-β_1,_ hUMSC human umbilical cord-derived mesenchymal stem cells, α-SMA alpha smooth muscle actin, Cyp17a1 Cytochrome P450 17A1, qRT-PCR quantitative real-time polymerase chain reaction, GAPDH glyceraldehyde-3-phosphate dehydrogenase, and SD standard deviation

The characterization of isolated ovarian stromal cells was confirmed by immunofluorescence staining with vimentin, cytokeratin, and factor VIII [33]. As shown in Fig. 5b, the cells were positively stained with vimentin but not cytokeratin and factor VIII. We investigated whether hUMSC transplantation affects stromal cell differentiation through the TGF-β_1_ signaling pathway. In order to find the appropriate concentration of CDDP and TGF-β_1_ inhibitor SB431542 to treat stromal cells, according to previous reports, the CCK-8 kit was used to detect the viability of stromal cells at different concentrations [34–36]. The CDDP concentration at 20 μM was selected since the treatment resulted in the cell viability as 82.82% (Fig. 5c). And the TGF-β_1_ inhibitor SB431542 concentration at 10 μM was selected since the treatment resulted in the cell viability as 105.24% (Fig. 5d). The immunofluorescence staining (Fig. 5f) and qRT-PCR (Fig. 6k) showed that the expression of Cyp17a1, a marker of TCs, was decreased in the CDDP-induced POI group. While the expression of α-SMA, a marker of MFB, was increased. Following the treatment with hUMSC medium and TGF-β_1_ inhibitor SB431542, the images showed an increase of Cyp17a1 expression and a decrease of α-SMA expression in hUMSCs + POI and POI + SB431542 groups (Fig. 5e–g). The western blot and qRT-PCR analysis showed that the expression of TGF-β_1_ and p-smad3 was increased in the POI group compared to the control group. Following the hUMSC medium treatment, the increased expression of TGF-β_1_ and p-smad3 was reversed in both protein and RNA levels. The treatment with TGF-β_1_ inhibitor also resulted in similar results as hUMSCs shown in Fig. 6. In summary, these data suggested that the TGF-β_1_/Smad3 signaling pathway was involved in the differentiation of stromal cells into TCs and subsequently inhibit the fibrosis of ovarian tissues in POI rats.

## Discussion

Stem cell transplantation has broad application prospects in repairing damaged tissues. According to reports, stem cell therapy can be used as a powerful tool to restore fertility and pregnancy [37]. Among the stem cells, the hUMSCs isolated from the human umbilical cord gained great attention due to its availability, T immunodeficiency, and multipolarity differentiation potential [15, 38, 39]. In our previous study, we demonstrated that hUMSC transplantation can restore zona pellucida glycoprotein 3 (pZP3)-induced ovarian function damage [29]. However, it is unclear whether hUMSC transplantation can restore the ovarian damage induced by chemotherapy drugs in POI rats. Therefore, the purpose of our research is to determine whether MSCs derived from the human umbilical cord can restore CDDP-induced ovarian function damage. Furthermore, we have explored its possible mechanism of repairing ovarian injury.

Ovarian tissue fibrosis is a basic pathological change of POI. Studies have shown that TGF-β_1_ signaling pathways are involved in fibrosis of multiple organs. To explore the mechanisms of the recovery of ovarian tissues following hUMSC implantation in POI rats, we have investigated whether hUMSC transplantation can inhibit ovarian fibrosis through the TGF-β_1_ signaling pathway. The POI rat model was successfully established following the chemotherapy drug CDDP treatment as reported [12, 24]. Following the hUMSC transplant, the damaged ovarian function of POI rats was significantly improved based on the changes of serum sex hormones such as E_2_, FSH, and LH. At the same time, the follicle numbers at each developmental stage and fertility ability have significantly increased in POI rats following hUMSC transplantation. These results suggest that the hUMSC transplantation has successfully restored the ovarian function in CDDP-induced POI rats. The results are consistent with the results of a previous study [40].

The basic pathological changes of POI include ovarian tissue fibrosis and follicular development disorders [5, 6]. In the ovary, stromal cells are an important component of the tissue and can differentiate into theca cell layer, including inner TCs and outer MFB [17–19]. Some studies have shown that fibrosis is closely related to MFB. The possible mechanism is that MFB effectively secretes large amounts of ECM and eventually leads to organ fibrosis [41–43]. The results from our study demonstrated that the hUMSC transplantation could reduce the ovarian tissue fibrosis in POI rats. The decreased expression of α-SMA and collagen in the in vitro cell culture study in the hUMSC treatment group may help explain its mechanism to inhibit the ovarian tissue fibrosis in POI rats. These results suggest that hUMSC transplantation restores ovarian function in POI rats by improving ovarian stromal cell differentiation to reduce the ovarian fibrosis.

TGF-β_1_ signaling pathway mediated by Smad protein plays an important role in the development of tissue fibrosis [44]. Recent studies have shown that the TGF-β_1_/Smad3 signaling pathway is involved in fibrosis in many organs [45, 46]. In our study, the data showed that the TGF-β_1_/Smad3 signaling was involved in ovarian tissue fibrosis in POI rats. The results are consistent with those studies that have demonstrated that the TGF-β_1_ pathway plays an important role in the pathogenesis of tissue fibrosis. Following the hUMSC transplantation, the ovarian fibrosis in POI rats was significantly reduced. These findings were further confirmed by blocking the collagen production in cultured stromal cells with TGF-β_1_ inhibitor SB431542 treatment. Also, the stromal cells co-cultured with CDDP and SB431542 were differentiated into TCs with less MFB presence. All these in vitro and in vivo experiment findings suggest that that TGF-β_1_/Smad3 signaling plays an important role in the recovery of ovarian function through its inhibition of tissue fibrosis in POI rats following hUMSC transplantation. This provides useful information for the development of pharmaceutical therapy treatment methods for POF failure in the future.

## Conclusion

The results from our study showed that hUMSC transplantation can recover the function of ovarian function in POI rats. This recovery is associated with the inhibition of ovarian fibrosis in part through regulating stromal cell differentiation via the TGF-β_1_/Smad3 signaling pathway. With the increasing number of POI patients with chemotherapy treatment, the results of this study provide new therapeutic targets and strategies for promoting the repair and recovery of ovarian function in such patients.

## Supplementary information

**Additional file 1: Supplementary Figure 1.** Flowcytometria analysis of cell markers in hUMSCs. The hUMSCs were characterized by its cell surface marker expression to confirm its phenotype. [a-g]: The following surface markers were identified: CD44, CD90, CD73, CD45, CD34, HLA - DR and CD105. [h-i]: The hUMSCs staining with Alizarin red S and Oil Red O to confirm its differentiation to Osteoblasts (100×) and adipoblasts (200×). [j, 400×]: The hUMSCs shows fibroblast - like morphology under light microscopy examination. hUMSCs human umbilical cord-derived mesenchymal stem cells.

## Data Availability

All data generated and/or analyzed during this study are included in this published article.

## Abbreviations

POI: Primary ovarian insufficiency
hUMSCs: Human umbilical cord-derived mesenchymal stem cells
TGF-β_1_: Transforming growth factor-β_1_
CDDP: Cisplatin
ELISA: Enzyme-linked immunosorbent assay analysis
Cyp17a1: Cytochrome P450 17A1
α-SMA: Alpha smooth muscle actin
qRT-PCR: Quantitative real-time polymerase chain reaction
E_2_: Estradiol
HRT: Hormone replacement therapy
MSCs: Mesenchymal stem cells
TCs: Theca cells
MFB: Myofibroblast
ECM: Extra-cellular matrix
PCOS: Polycystic ovary syndrome
DW: Distilled water
PBS: Phosphate buffer saline
FBS: Fetal bovine serum
FCM: Flow cytometry
GCs: Granulosa cells
FSH: Follicle-stimulating hormone
LH: Luteinizing hormone
HE: Hematoxylin and eosin
GAPDH: Glyceraldehyde-3-phosphate dehydrogenase
SDS–PAGE: Sodium dodecyl sulfate polyacrylamide gel electrophoresis
PVDF: Polyvinylidene fluoride
TBST: TBS added with Tween 20
ECL: Enhanced chemiluminescence reagent
DAPI: 4,6-Diamino-2-phenyl indole
SD: Standard deviation
ANOVA: One-way analysis of variance
Th1: T-helper 1
Th2: T-helper 2
pZP3: Zona pellucida glycoprotein 3
